# Persistent atrial fibrillation originating from prominent Eustachian ridge: Precise identification of non–pulmonary vein foci using a high-density grid mapping catheter

**DOI:** 10.1016/j.hrcr.2021.03.008

**Published:** 2021-03-18

**Authors:** Yasuteru Yamauchi, Rena Nakamura, Takatoshi Shigeta, Yuichiro Sagawa, Kaoru Okishige, Tetsuo Sasano

**Affiliations:** ∗ Department of Cardiology, Japan Red Cross Yokohama City Bay Hospital, Yokohama City, Japan; † Department of Cardiovascular Medicine, Tokyo Medical and Dental University, Tokyo, Japan

**Keywords:** Atrial fibrillation, Eustachian ridge, High-density grid mapping catheter, Inferior vena cava, Intra-atrial conduction block, Non–pulmonary vein, Self-reference mapping

## Introduction

Atrial fibrillation (AF) mostly originates from the pulmonary vein (PV). As a result, 70%−90% of AF patients can be successfully treated with extensive PV isolation. However, the precise identification of the site of trigger in the remaining 10%–30% with non-PV foci^1^, ^2^, ^3^ is often difficult. Especially in cases that require multiple electrical cardioversions because of immediate recurrence of AF after cardioversion, the mapping of origins becomes more difficult. In this case, a prominent Eustachian ridge was demonstrated as a source of AF trigger by high-density grid mapping catheter (Advisor™ HD Grid Catheter; Abbott Medical, Minneapolis, MN).

## Case report

**Figure 1:**
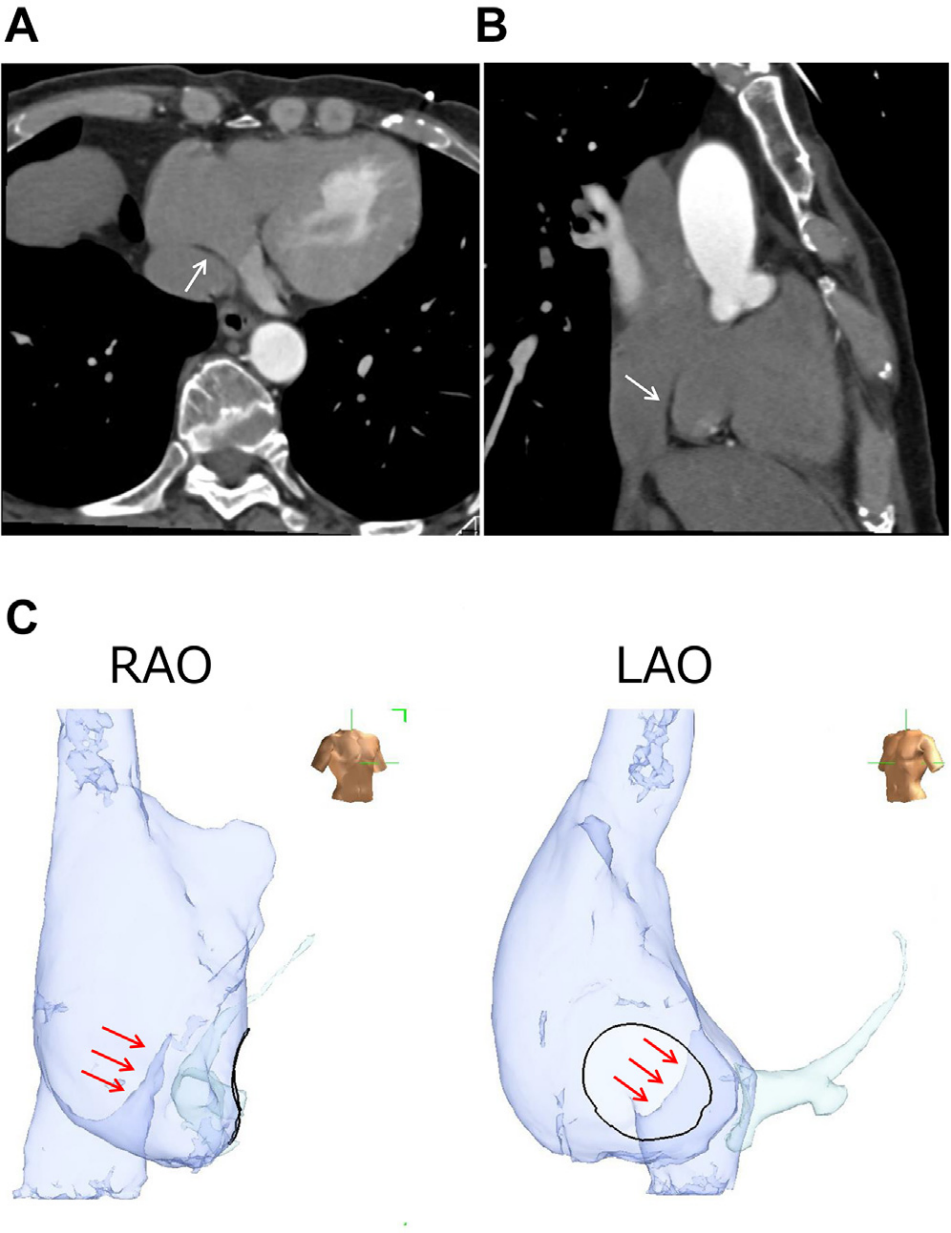
Computed tomography (CT) images. **A:** Axial view. **B:** Sagittal view. **C:** Three-dimensional CT image of cavotricuspid isthmus level. The prominent Eustachian ridge (*white and red arrows*) is noted. LAO = left anterior oblique; RAO = right anterior oblique.

**Figure 2:**
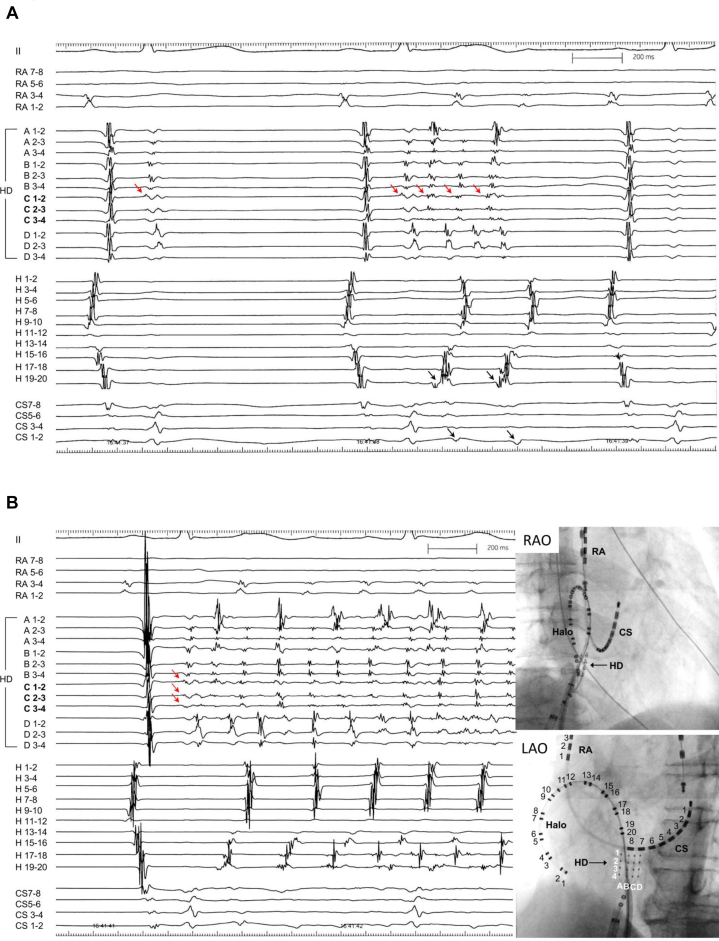
Intracardiac electrograms immediately after electrical cardioversion. A high-density grid catheter was situated at the prominent Eustachian ridge. **A:** A single atrial ectopic beat was recorded only at the high-density grid catheter. However, this excitement was not conducted to the surrounding right atrium and the coronary sinus, suggesting an intra-atrial conduction block. Then, 4 consecutive ectopic beats were conducted in the surrounding right atrium and the coronary sinus in a 2:1 fashion. **B:** A few seconds later, atrial ectopic beats originating from the Eustachian ridge triggered atrial fibrillation. The earliest atrial activation was recorded at the C-spline of the high-density grid catheter (not A- or D-spline), suggesting the precise identification of these non-PV foci. CS = coronary sinus; H = halo duodecapolar catheter; HD = high-density grid catheter; LAO = left anterior oblique; RA = high right atrium; RAO = right anterior oblique.

**Figure 3:**
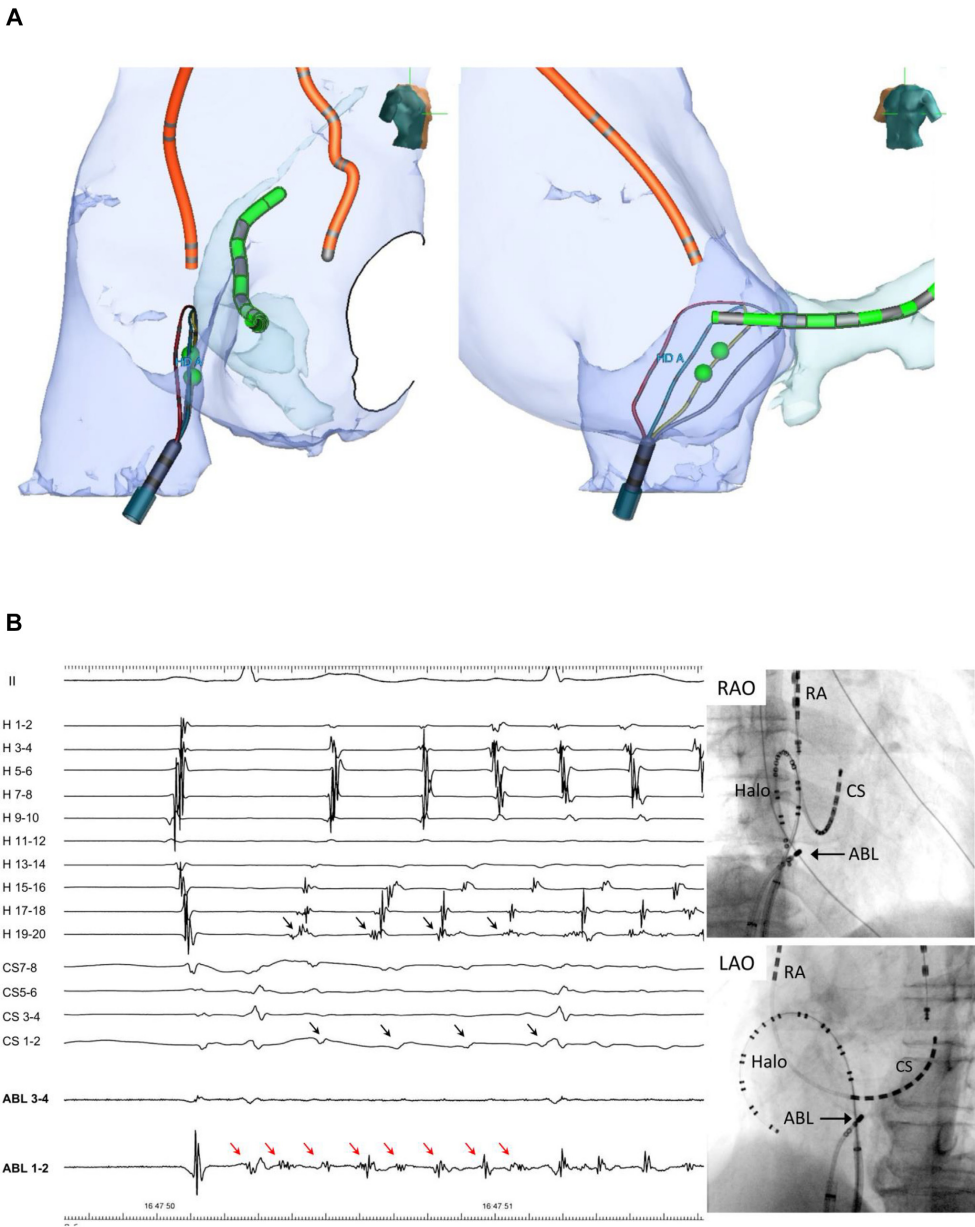
The earliest atrial activation site during atrial fibrillation (AF) initiation. **A:** A 3-D mapping system revealed that a high-density grid catheter was situated at the prominent Eustachian ridge. The green tag was marked at the earliest atrial activation site of the C-spline of the high-density grid catheter. **B:** After the high-density grid catheter was pulled out, the ablation catheter was positioned at the green tag sites. Intracardiac electrogram of the distal pair of the ablation catheter was the earliest activation site during AF initiation. A couple of radiofrequency applications were performed. ABL = ablation catheter; CS = coronary sinus; H = halo duodecapolar catheter; LAO = left anterior oblique; RA = high right atrium; RAO = right anterior oblique.

A 50-year-old man was referred for catheter ablation owing to persistent AF. Echocardiography showed a normal ejection fraction and a left atrial diameter of 44 mm. After PVs and left atrial posterior wall (LAPW) isolation were performed during AF, electrical cardioversion converted AF to sinus rhythm. However, after a few seconds, AF recurred. Despite repeated electrical cardioversions, AF recurred in the same manner. Then, we placed a wide halo catheter with duodecapolar electrodes in the whole right atrium and a ring catheter at the left atrial septum so as to map the right atrial free wall and both atrial septums simultaneously. The sequence of the first beat triggering AF was always the same, showing the origin close to the lower right atrial septum. Using a high-density grid catheter, the site of origin was mapped precisely with a minimum number of cardioversions, as shown in the Supplemental Figure. The earliest activation site was depicted in the anterior direction of the right atrium in this way. An enhanced computed tomography (CT) scan image recorded before the procedure revealed a prominent Eustachian ridge (Figure 1) at that area. Therefore, the high-density grid catheter was placed towards this prominent Eustachian ridge. After cardioversion, a single atrial ectopy occurred with a short interval of 170 ms conducting to the surrounding right atrium in 2:1 fashion, suggesting the presence of an intra-atrial conduction block (Figure 2A). A few seconds later, local ectopic beats with rapid firing of a mean cycle length of 120 ms triggered sustained AF. Furthermore, the earliest activation site of the first ectopic beat was the C-spline of the high-density grid catheter, neither the A-spline nor the D-spline. This finding implied that the exact non-PV foci were situated within this high-density grid catheter facing the prominent Eustachian ridge. After tagging the site of earliest activation (2 and 3 of the C-spline, Figure 3A), a radiofrequency catheter was placed at the same area. After cardioversion, similar high-frequency activation inducing AF was recognized by the radiofrequency catheter (Figure 3B). After a couple of ablations at the site, AF did not occur even after cardioversion. AF could not be induced by any atrial drive train with high doses of isoproterenol. The patient was free from AF without any antiarrhythmic drugs during 18 months of follow-up.

## Discussion

The sites of non-PV foci triggering AF are mainly the superior vena cava (SVC), LAPW, and left atrium around the PV.^1^ The other sites include the vein of Marshall, coronary sinus, atrial septum, mitral annulus, tricuspid annulus, both atrial appendages, and the inferior vena cava (IVC).^2^ Non-PV foci originating from the Eustachian ridge is reportedly extremely rare. Hayashi and colleagues^3^ reported the distribution of 39 mappable non-PV foci and favorable ablation outcomes of this mappable non-PV AF. Of the 39 non-PV foci, 15 and 6 originated from the SVC and LAPW, respectively.^3^ These non-PV foci are easily curable with SVC and LAPW isolation, and therefore, precise identification is not always necessary. However, regarding other non-PV foci, precise identification of the foci is necessary for obtaining successful ablation. However, conventional mapping methods have some limitations. In the present study, we used a high-density grid catheter to map the exact origin utilizing its structure with both vertical and horizontal splines. A self-reference mapping was performed by tagging the earliest activation site of a high-density grid catheter on a 3-dimensional mapping image and then moving the catheter to the upstream of the excitation one after another (Supplemental Figure). Finally, the site of earliest activation could be precisely depicted with a minimum number of cardioversions as possible. This method could be done relatively easily by an ultra-high-density mapping catheter. The Eustachian ridge is a normal anatomical structure that separates the orifice of the IVC from that of the coronary sinus. Waki and colleagues^4^ reported that the pectinate muscles radiated from the crista terminalis in the cavotricuspid isthmus in a parallel fashion, and the Eustachian ridge contained a muscular extension from the crista terminalis in most cases. Cabrera and colleagues^5^ also demonstrated that 8 of 30 (27%) heart specimens had a prominent Eustachian ridge with a thick musculature. A histological study demonstrated the presence of pacemaker cells within the Eustachian ridge, suggesting a potential source of abnormal automaticity causing atrial tachycardia or AF.^6^ In this patient, local ectopic beats with intra-atrial conduction block, similar to PV firing with exit block in patients with PV-originated AF, were observed. The presence of the Eustachian ridge is difficult to determine by conventional right atrial angiography alone; therefore, preoperative contrast-enhanced CT or intracardiac echocardiographic evaluation is very useful. Although some authors reported AF originating from the IVC,^7^^,^^8^ Igawa and colleagues^9^ reported that IVC extension into the posteroinferior right atrium exists and varies, although this site always lacks myocardium on a detailed anatomical assessment of the IVC and the right atrial junction. Based on these findings, AF originating from the Eustachian ridge may be misdiagnosed as that originating from the IVC if the anatomy is not thoroughly evaluated with contrast CT or other methods.

## Conclusion

A high-density grid mapping catheter could be a useful tool for precise identification of non-PV foci. Prominent Eustachian ridge might be an important source of non-PV foci, which could be misunderstood as a different source.

## Appendix Supplementary data

Supplemental Figure A self-reference mapping using a high-density mapping catheter. The earliest activation site of a high-density grid catheter was tagged on a three-dimensional mapping image and then a high-density grid mapping catheter was moved upstream of the excitation one after another in order of (A), (B), (C), (D). Finally, the site where the tagged earliest activation site overlaps the previous tagged site was exactly the precise origin of tachycardia at the non-PV foci triggering AF in (E) and (F).
