# Supplementary material for: Persistent atrial fibrillation originating from prominent Eustachian ridge: Precise identification of non–pulmonary vein foci using a high-density grid mapping catheter
Source: HeartRhythm Case Rep. 2021 Mar 18;7(6):386–90. doi: 10.1016/j.hrcr.2021.03.008 (PMC8226311; doi:10.1016/j.hrcr.2021.03.008)
Supplement: Supplemental Figure — A self-reference mapping using a high-density mapping catheter. The earliest activation site of a high-density grid catheter was tagged on a three-dimensional mapping image and then a high-density grid mapping catheter was moved upstream of the excitation one after another in order of (A), (B), (C), (D). Finally, the site where the tagged earliest activation site overlaps the previous tagged site was exactly the precise origin of tachycardia at the non-PV foci triggering AF in (E) and (F). [file mmc1.docx]

Supplemental Figure legend.

A self-reference mapping using a high-density mapping catheter. The earliest activation site of a high-density grid catheter was tagged on a three-dimensional mapping image and then a high-density grid mapping catheter was moved upstream of the excitation one after another in order of (A), (B), (C), (D). Finally, the site where the tagged earliest activation site overlaps the previous tagged site was exactly the precise origin of tachycardia at the non-PV foci triggering AF in (E) and (F).
