# Supplementary figures and images for: Persistent atrial fibrillation originating from prominent Eustachian ridge: Precise identification of non–pulmonary vein foci using a high-density grid mapping catheter
Source: HeartRhythm Case Rep. 2021 Mar 18;7(6):386–90. doi: 10.1016/j.hrcr.2021.03.008 (PMC8226311; doi:10.1016/j.hrcr.2021.03.008)

## Slide 1
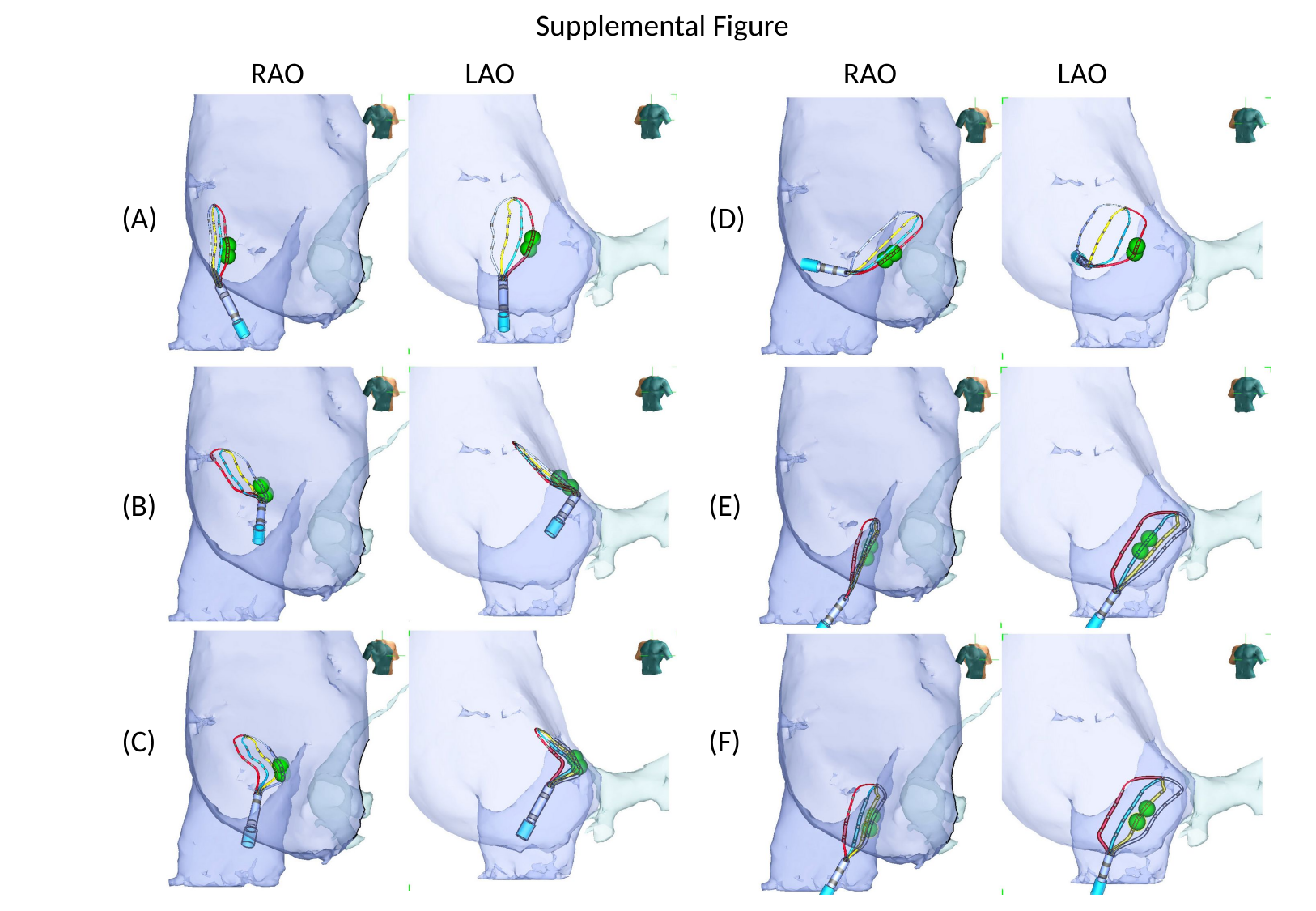

Supplemental Figure
RAO
LAO
RAO
LAO
(A)
(D)
(B)
(E)
(C)
(F)

Supplement: Supplemental Material [file mmc2.pptx]
